# Effect of age and season on the thyroid hormone activity of Mizoram strain female mithun (*Bos frontalis*)

**DOI:** 10.14202/vetworld.2015.1375-1378

**Published:** 2015-12-11

**Authors:** Lalsangpuii, M. Ayub Ali, L. Inaotombi Devi, Parthasarathi Behera, Lalsanglura Ralte

**Affiliations:** 1 Department of Veterinary Physiology and Biochemistry, College of Veterinary Sciences and A.H., Central Agricultural University, Selesih, Aizawl, Mizoram, India; 2 Department of Medical Laboratory Technology, Regional Institute of Paramedical and Nursing Sciences, Mizoram, India

**Keywords:** age, mithun, triiodothyronine, thyroxine, thyroid hormone, season

## Abstract

**Aim:**

The aim of the present study was to generate baseline data on the normal values of the thyroidhormone (TH) activity as well as their correlation with age and season.

**Materials and Methods:**

Blood samples (10 ml) were collected from jugular vein of 30 female mithun’s of three different age groups *viz*. Calves (6 months to 1 year), heifer (1-3 years) and adult (above 3 years) during the three season’s viz. Monsoon, winter and spring of a year. The serum was analyzed for thyroid stimulating hormone (TSH), triiodothyronine (T_3_), and thyroxine (T_4_) activity.

**Result:**

The result showed a significantly (p<0.05) a higher T_3_ level in heifers followed by adults and calves and higher T_4_ level in adults followed by heifers and calves in all the seasons. The TSH level was higher in heifers in all the seasons. The winter season recorded higher level of T_3_, T_4_, and TSH as compared to the other seasons of a year.

**Conclusion:**

The TSH and T_3_ level were the highest for aheifer, whereas T_4_ level was the highest for adults inall the season. Furthermore, the higher level of TH was observed in winter season. The increased level of the TH during the winter season signifies their calorigenic effect. Similarly in heifers, the increased T_3_ concentrations show its importance in reproductive physiology and its association with ovarian activity. This indicates that age and season have aprofound effect on TH activity of Mizoram strain female mithun.

## Introduction

Mithun (*Bos frontalis*), is a semi-wild ruminant found in the north-eastern hilly regions of India besides Myanmar, Bhutan, Bangladesh, China, and Malaysia. They are the mainstay of meat production system in the north-eastern India. This bovine species is believed to be domesticated for more than 8000 years [1] from wild Gaur (*Bos gaurus*). Mizoram is home to 0.73% (1939) of mithun in India (18^th^ Livestock census 2007) and is mostly concentrated in Champhai and Saiha districts.

The thyroid gland, one of the largest endocrine glands, plays an important role in the body metabolism through its secretions *viz*. triiodothyronine (T_3_) and thyroxine (T_4_). These thyroid hormones (TH) are iodinated derivatives of the amino acid ­tyrosine [2] and are involved in the metabolic response of animals to certain nutritional, environmental and/or ­disease-relatedchallenges, as well as in regulation of certain ovarian functions[3]. However, the majority of T_3_ is derived from partial deiodination of T_4_ rather than the thyroid gland, and thus T_3_ is considered biologically more active than T_4_ [4]. In animals, TH is required for normal growth and development indicating a pivotal role in growth regulation. Since they are necessary for normal growth; optimal concentrations of TH act as growth stimulators [5].

The thyroid hormones are the central regulators of energy metabolism [6]. These hormones are the primary endocrine stimulators of non-shivering (“facultative” or “adaptive”) thermogenesis, thus regulating body temperature. They stimulate expression and activity of uncoupling proteins (UCPs), which uncouple re-oxidation of reduced coenzymes to ADP phosphorylation, hence producing heat [7]. A major exogenous regulator of thyroid gland activity is the environmental temperature. During heat stress, blood T_3_, and T_4_ concentrations, as well as metabolic rate, feed intake, growth and milk production are decreased [8-10]. The seasonal pattern of blood TH levels often showed maximal values during winter (cold months) and minimal during summer (hot months) [11,12]. Seasonal variation in plasma concentration of TH was reported in camel [6], cattle [13], buffaloes [12,14], and in goats [15].

Until date, only a few studies have been documented regarding hormonal profile [16,17] of mithun. However, to the best of our knowledge there are no previous reports regarding the effect of age and season on TH activity of Mizoram strain female mithun. Keeping this in view, the present study was done to find out the serum TH activity and their relationship with age and season.

## Materials and Methods

### Ethical approval

The study was carried out after the approval from Institute Animal Ethics Committee (IAEC) of College of Veterinary Sciences & Animal Husbandry, Central Agricultural University, Aizawl, Mizoram.

### Sample collection

A total of 90 blood samples (10 blood samples each) of female mithun at different age groups *viz*. Calves (6 months to <1 year of age), heifers (from 1 year to <3 years), and adults (3 years and above) were collected at random from different mithun farmers’ holdings located in Champhai and Saiha districts of Mizoram. The blood samples were collected in three different seasons (monsoon, winter, and spring) of a year. Approximately, 10 ml venous blood from each mithun was collected from jugular vein using syringes with 18G 1.5 hypodermic needles.

### Serum extraction and processing

Immediately after collection, the blood samples were transferred into 15 ml centrifuge tubes without anticoagulant and kept in a slanting manner for 1 h at room temperature for coagulation. The serums were separated by centrifugation at 2500 rpm for 10 min and collected in sterile screw capped cryo-vials immediately and were transported to the research laboratory of Veterinary Physiology and Biochemistry Department, College of Veterinary Sciences and A.H., CAU, Selesih, Aizawl on ice. The serum sample were processed immediately for TSH, T_3_, and T_4_ level by a MicroplateImmunoenzymometric Assay method using diagnostic kit (M/s RFCL Limited, Dehradun) as per the manufacturer’s instruction by measuring the absorbance at 450 nm in a Thermo ELISA plate reader.

### Statistical analysis

The data were analyzed by ANOVA and were found to be statistically significant at p<0.05.

## Results

**Figure-1:**
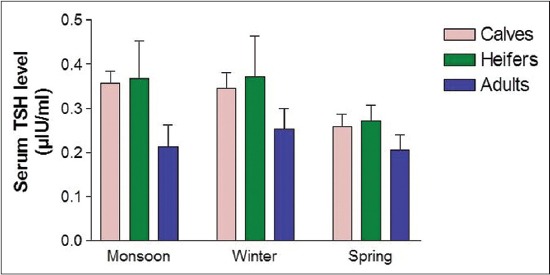
Serum thyroid stimulating hormone level at different age and seasons.

**Figure-2:**
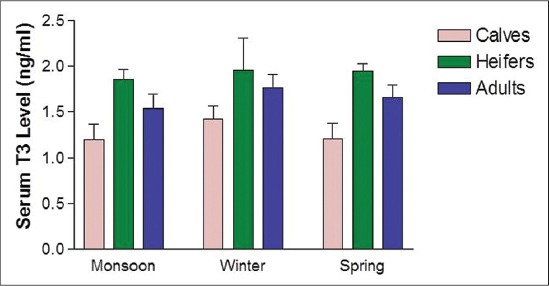
Serum triiodothyronine level at different age and seasons.

**Figure-3:**
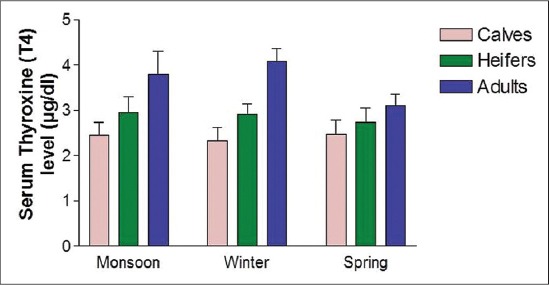
Serum thyroxine level at different age and seasons.

The concentrations of serum thyroid-stimulating hormones (TSH), T_3_ and T_4_ are presented in Table-1. The levels of serum TSH, T_3_, and T_4_ differed significantly among the different age groups. Critical difference (CD) test revealed that significantly highest TSH level (0.34±0.09) was observed in heifer followed by calves and the lowest value (0.22±0.05) in adults (Figure-1) and for T_3_, significantly highest value (1.92±0.21 ng/dl) was observed in heifers, whereas, the calves depicted the lowest value (1.27±0.19 ng/dl) (Figure-2). However, the serum T_4_ level was found to increase with theage of the animal in all seasons. The serum T_4_ level ranged between 2.32±0.30 (µg/dl) and 4.08±0.29 (µg/dl), and the significantly highest level (3.66±0.55 µg/dl) was observed in adults, whereas the lowest value (2.41±0.30 µg/dl) was observed in calves (Figure-3).

**Table-1:** Serum TSH, T_3_, and T_4_ levels at different age and different seasons.

Season	Age	Parameters		

		TSH (µIU/ml)	T_3_ (ng/ml)	T_4_ (µg/dl)
Monsoon	Adult	0.22±0.05^bB^	1.54±0.15^aB^	3.66±0.55^aA^
	Heifer	0.37±0.09^aA^	1.92±0.21^A^	2.95±0.35^B^
	Calves	0.36±0.03^aA^	1.27±0.19^bC^	2.45±0.29^C^
Winter	Adult	0.25±0.05^aB^	1.77±0.14^aA^	4.08±0.29^aA^
	Heifer	0.37±0.09^aA^	1.96±0.35^A^	2.90±0.23^B^
	Calves	0.35±0.04^aA^	1.42±0.15^aB^	2.32±0.30^C^
Spring	Adult	0.21±0.03^bB^	1.66±0.14^aB^	3.11±0.24^bA^
	Heifer	0.27±0.04^bA^	1.95±0.08^A^	2.740±0.31^B^
	Calves	0.26±0.03^bA^	1.21±0.16^bC^	2.41±0.30^C^

TSH=Thyroid stimulating hormone, T_3_=Triiodothyronine, T_4_=Thyroxine, The means with different superscript in capital letters and small letters differs significantly (p<0.01) between the rows and columns

## Discussion

The TH play a key role in co-ordination of different factors involved in growth, which is of great economic importance as far as the livestock enterprises are concerned [5]. TH directly influence growth by altering biochemical reactions; and being anabolic hormones, cause positive nitrogen balance and promote growth and development [5]. In the present study, the TSH levels were lowest in calves and increases among the heifer and the level decreases in the adult. This shows that the TSH synthesis is inhibited by increasing circulatory levels of T_3_ and T_4_ in their respective ages. The synthesis of TH is regulated by feedback regulation. The increased synthesis of TSH occurs in response to decreased circulatory levels of T_3_ and T_4_. Among the different seasons of sample collection, the highest level of TSH was observed during monsoon and winter in all the age groups while the activity was decreased during spring. Similarly, the highest level of T_3_ was observed during the winter season. Seasonal variation in the heifers, however, was non-significant and for T_4_, the highest level was observed during the winter season. Bhullar *et al*. [18] have also reported the variation in plasma T_3_ concentration in buffalo which is in agreement with our findings. Similar findings regarding T_3_ level have been reported by Mayahi [11] in buffalo. The findings of Polat *et al*. [19] is also in line with our findings who observed decrease in T_3_ and T_4_ level with increase in temperature in case of white goats. The cold environment could be a stimulus to increase the thyrotrophic hormone output thereby resulting in a higher concentration of TH in serum. Zhang *et al*. [10] reported that during heat stress there was asignificant reduction in concentrations of T_3_ and T_4_ in plasma and in milk of lactating cows. The highest concentration of T_3_ was observed in heifers and then decreasing with advancing age in female Mithun is in accordance with Garg *et al*. [20]. The higher concentration of T_3_ in heifer could be one of the adaptive mechanisms to overcome the stressful period and subsequent declining trend could be attributed to the negative feedback mechanism exerted by already higher concentrations of T_3_ in blood. Besides, this increase in T_3_ secretion may also be due to higher TSH concentration or decreased T_3_ metabolic clearance due to low capability of T_3_ degrading enzymatic system in heifer. The serum T_4_ level was found to increase with age of the animals in all seasons. The serum T_4_ level in the present investigation ranged between 2.32±0.30 and 4.08±0.29 µg/dl. The overall mean values were found to differ significantly between the different age groups by applying CD test that revealed significantly the highest mean value in adults, followed by heifer and the lowest being in calves. The results are also in line with the finding of Ingole *et al*. [5] who reported a positive relationship between circulatory level of TH with age in buffaloes and in Jamunapari breeds of Goat [21]. The observed increased level of the TH during the winter season signify the calorigenic effect and the processes and pathways mediating the intermediary metabolism of carbohydrates, lipids, and proteins are all affected by THs. Similarly in heifers, the increased T_3_ and T_4_ concentrations shows its importance in reproductive physiology and its association with ovarian activity. The slight high levels of T_4_ during prepubertal stage may be necessary for theenhanced synthesis of protein and gain in weight.Similar to the present study, Gray *et al*. [22] reported increase in T_4_ concentrations with age which is required precisely in regulated amounts for normal tissue growth process. Furthermore, the increase T_4_ levels have been proposed as one of the modes of action of estrogen to bring to cyclicity [20]. Moreover, Refesal *et al*.[23] observed in cows a significant herd and season interaction for both the T_3_ and T_4_ hormones. Similar to T_3_, Rasooli *et al*. [24] reported that in Holstein heifer, the serum concentration of T_4_ in summer was lower than in winter. T_4_ is a calorigenichormone and the thyroid activity is enhanced for maintaining constant body temperature by increasing the metabolic rate when exposed to thecold environment.

## Conclusions

It can be concluded from the present study that the TSH and T_3_ level were highest for heifer whereas T_4_ level was the highest for adults inall the season. The increased level of the THs during the winter season signifies their calorigenic effect. Similarly in heifers, the increased T_3_ concentrations show its importance in reproductive physiology and its association with ovarian activity.

## Authors’ Contributions

LSP and MAA designed the experiment. LSP, MAA, and LID conducted the experimental work. LSP, MAA, PB and LSR were involved in scientific discussion and analysis of the data. LSP, MAA, and PB drafted and revised the manuscript. All authors read and approved the final manuscript.
